# Striatal transcriptome of a mouse model of ADHD reveals a pattern of synaptic remodeling

**DOI:** 10.1371/journal.pone.0201553

**Published:** 2018-08-15

**Authors:** Anastasia M. Sorokina, Michael Saul, Tassia M. Goncalves, Joseph V. Gogola, Petra Majdak, Sandra L. Rodriguez-Zas, Justin S. Rhodes

**Affiliations:** 1 Department of Psychology, Beckman Institute for Advanced Science and Technology, University of Illinois, Urbana, Illinois, United States of America; 2 Institute for Genomic Biology, University of Illinois, Urbana, Illinois, United States of America; 3 Department of Animal Sciences, University of Illinois, Urbana, Illinois, United States of America; 4 Department of Psychology, University of Chicago, Chicago, Illinois, United States of America; 5 The Neuroscience Program, University of Illinois, Urbana, Illinois, United States of America; Technion Israel Institute of Technology, ISRAEL

## Abstract

Despite the prevalence and high heritability of Attention-Deficit/Hyperactivity Disorder (ADHD), genetic etiology remains elusive. Clinical evidence points in part to reduced function of the striatum, but which specific genes are differentially expressed and how they sculpt striatal physiology to predispose ADHD are not well understood. As an exploratory tool, a polygenic mouse model of ADHD was recently developed through selective breeding for high home cage activity. Relative to the Control line, the High-Active line displays hyperactivity and motor impulsivity which are ameliorated with amphetamine. This study compared gene expression in the striatum between Control and High-Active mice to develop a coherent hypothesis for how genes might affect striatal physiology and predispose ADHD-like symptoms. To this end, striatal transcriptomes of High-Active and Control mice were analyzed after mice were treated with saline or amphetamines. The pseudogene *Gm6180* for n-cofilin (*Cfl1*) displayed 20-fold higher expression in High-Active mice corresponding with reduced *Cfl1* expression suggesting synaptic actin dysregulation. Latrophilin 3 (*Lphn3*), which is associated with ADHD in human populations and is involved in synapse structure, and its ligand fibronectin leucine rich transmembrane protein 3 (*Flrt3*), were downregulated in High-Active mice. Multiple genes were altered in High-Active mice in a manner predicted to downregulate the canonical Wnt pathway. A smaller and different set of genes including glyoxalase (*Glo1)* were differentially regulated in High-Active as compared to Control in response to amphetamine. Together, results suggest genes involved in excitatory synapse regulation and maintenance are downregulated in ADHD-like mice. Consistent with the molecular prediction, stereological analysis of the striatum from a separate set of mice processed for imunohistochemical detection of synaptophysin revealed approximately a 46% reduction in synaptophysin immunoreactivity in High-Active relative to Control. Results provide a new set of molecular targets related to synapse maintenance for the next generation of ADHD medicines.

## Introduction

Attention Deficit Hyperactivity Disorder (ADHD) is a widespread disorder with approximately 4.8% of children in the United States receiving medication for ADHD symptoms. Despite its prevalence, the long-term impact of medications commonly used to treat ADHD in humans–*e*.*g*., amphetamines (Adderall) and methylphenidate (Ritalin)–on the developing brain is not well known, and many studies demonstrate a high potential for abuse [1–3]. An improved understanding of the biological basis for ADHD is crucial for finding new targeted drugs and treatments that would alleviate the common symptoms of ADHD, such as hyperactivity and impulsivity, while minimizing unwanted side effects.

Twin and adoption studies indicate that the disorder is highly heritable, with heritability estimates ranging from 70–90% [4], suggesting an outsized role for genetics in the etiology of ADHD. Several genes have been identified from genome wide association studies (GWAS), but together they account for only a small proportion of the heritability [5,6]. Current molecular and candidate gene-association studies of ADHD have focused mainly on catecholamine systems, in part because of the hypothesized targets of amphetamine and methylphenidate on dopaminergic and noradrenergic systems [7]. An alternate possibility is that catecholamine system dysregulation is a symptom of a different mechanistic process.

Human MRI and fMRI studies have repeatedly identified striatum as displaying reduced gray matter volume and function in ADHD patients [8,9]. This alteration is apparent in both dorsal (caudate-putamen) and ventral (nucleus accumbens) portions of the striatum when comparing morphology and function between ADHD and control subjects. As compared to the other implicated brain regions such as the cerebellum and anterior cingulate cortex [10–12], the striatum receives the vast majority of dopaminergic projections from ventral midbrain nuclei including the substantia nigra and ventral tegmental area, supporting the catecholamine hypothesis. However, the connection between the specific genes implicated in ADHD and the re-wiring of the striatum towards an ADHD-like phenotype remains poorly understood.

For preclinical exploration and hypothesis generation, we developed a polygenic mouse model of ADHD using a selective breeding approach [13]. Our model includes a High-Active line, selectively bred for 17 generations for increased home cage activity and a Control, unselected line, maintained over the same period. Features of the model relevant for exploring ADHD etiology include polygenicity of the ADHD-like phenotypes, hyperactivity in a habituated environment, motor impulsivity, and symptoms ameliorated with therapeutic doses of amphetamines [13,14]. The goal of the present study was to document differences in gene expression between the High-Active and Control mice in the striatum and in response to amphetamines, in order to develop a more coherent hypothesis for how patterns of differentially expressed genes in the striatum might contribute to ADHD-like behavior.

Recent reports have implicated genes such as latrophilin 3 (*Lphn3*) and fibronectin leucine rich transmembrane protein 3 (*Flrt3*), which are involved in synaptic strength and structural maintenance, in the pathophysiology of ADHD. [15–17]. *Lphn3* is a member of the latrophilin subfamily of G-protein coupled receptors involved in adhesion between pre- and post-synaptic neuronal membranes. Interactions of Lphn3 with Flrt3 proteins are required for synapse development and function, specifically excitatory synapses [18]. Therefore, we hypothesized that *Lphn3*, *Flrt3* and other related genes that are involved in the development and maintenance of synapses may be downregulated, and possibly lead to decreased striatal excitatory synaptic density. However, beyond the genes and pathways previously implicated in ADHD, we had no prior hypotheses about which subset of genes might display such genotype-by-amphetamine interactions. Given the paradoxical effect of therapeutic doses of amphetamines on the symptoms reported previously [13], and the dopamine pharmacology of amphetamines, we expected to find genes that were oppositely regulated by amphetamine in the High-Active vs. Control genotypes.

## Materials and methods

### Experimental subjects

Young adult male mice (average age 6.5 months ± 0.80 SD) from generation 17 (Experiment 1) and generation 22 (Experiment 2) of a long term selective breeding experiment for increased home cage activity were used [13]. In brief, a starting population derived from a systematic inter-cross of 8 genetically variable inbred strains [19] was used as the founder strain for two mouse lines: High-Active and Control. In each generation mice were placed in custom cages which allow video tracking from above. Total distance traveled over days 5 and 6 of a 6-day test was used as the selection criterion. In the High-Active line, each generation, the top male and female from within each family were randomly bred with the only rule that sibling breeding was not allowed. In the Control line, a male and a female was randomly chosen to represent each family in the subsequent generation with the same rule avoiding sibling breeding. In experiment 1, a total of n = 10 High-Active, and n = 10 Control mice, each from a different family, were used. In experiment 2, a total of n = 6 High-Active and n = 6 Control mice were used.

## General husbandry

Rooms were kept at a constant temperature (21 ± 1 °C) and in a reversed 12:12 light-dark cycle with lights on at 7:30 pm and off at 7:30 am. Food and water were provided ad libitum (Harlan Teklad 7012). Corncob bedding (Harland Teklad 7097, Madison, Wisconsin, USA) was provided in all cages. All procedures were approved by the University of Illinois Institutional Animal Care and Use Committee and adhered to NIH guidelines. The Beckman Institute Animal Facility, where the experiments took place, is AAALAC approved.

### Experiment 1: RNA-seq

Mice were phenotyped as per previous work [13,14] for home cage activity when they were approximately 3 months old. At approximately 6.5 months of age, all the mice were re-phenotyped for home cage activity using the same procedure, 6 days of continuous video-tracking. On the 7^th^ day, 2 hours after the lights shut off in the animal facility, mice received an intraperitoneal (i.p.) injection (10 ml/kg) of either vehicle (0.9% saline) or amphetamine (0.25 mg/kg d-amphetamine sulfate in 0.9% saline, catalog number A-5880, Sigma-Aldrich, St. Louis, MO), and returned to their home cage for video tracking. Mice were euthanized exactly 2 hours following the injection by live decapitation. Acute response to single dose was used instead of chronic dosing, because effects of amphetamine on ameliorating symptoms of ADHD are not thought to result from neuroadaptations from chronic use, but rather direct acute psychoactive effects [20].

#### Striatum dissection

Following [21], all surfaces and instruments were cleaned thoroughly with RNAse away. Brains were rapidly dissected and immediately placed on an aluminum platform on wet ice with the ventral surface exposed. Using the olfactory tubercles and optic chiasm as landmarks, a razor blade was used to cut a coronal section approximately 1.7 mm thick containing the striatum. The section was then flipped onto its caudal aspect and then the entire bilateral striatum (dorsal and ventral) was carefully dissected from the cortex, medial septum, and olfactory tubercles. The striatum was then immediately placed in a centrifuge tube on dry ice and then stored at -80°C. The entire brain extraction and dissection process occurred over approximately 2 minutes.

#### RNA extraction and purification

Bilateral striatal tissue was homogenized with an RNase-free disposable pellet pestle (Thermo Fisher Scientific, Waltham, MA) and RNA was extracted using the commercially available RNeasy^®^ Lipid Tissue Mini Kit (Qiagen Inc., Valencia, CA). Purification of the isolated RNA included treatment with DNase I (Qiagen Inc, Valencia, CA), accordingly to the manufacturer’s instructions. For assessing total RNA yield, aliquot samples were measured with the Qubit^®^ 2.0 (Life Technologies). Each sample showed a 260/280 ratio over 2.0 and yielded over 14 μg of RNA. Quality and integrity of isolated RNA samples were determined by 28S/18S rRNA analysis with the Agilent 2100 Bioanalyzer (Santa Clara, CA). All samples scored an RNA Integrity Number (RIN) over 8, indicating no signs of degradation. Quality was spot checked by choosing random samples to run on a gel, which confirmed the Bioanalyzer report.

#### RNA sequencing

RNA-sequencing was performed by the Roy J. Carver Biotechnology Center. RNA-Seq libraries were prepared using the TruSeq Stranded RNA Sample Prep kit with an average fragment length of 200 bp. Libraries were quantified using qPCR, pooled in a pool of 20, and multiplexed across a total of 5 lanes. The libraries were sequenced on an Illumina HiSeq 2500 for 101 cycles in 100 bp paired-end format using a TruSeq SBS sequencing reagent kit, v. 4. Read depth ranged between 41,684,484 reads and 70,449,689 reads with a median read depth of 54,516,050 reads. FASTQ files were generated from the raw sequencing runs using CASAVA v. 1.8.2. Reads in FASTQ format were aligned to the Mus musculus Ensembl GRCm38 genome with Ensembl GRCm38.75 annotation using TopHat2 v. 2.0.10 22. Since downstream applications used a counts-based method, we counted reads mapping to genes in each sample using htseq-count from the HTSeq Python framework, v. 0.6.1 24.

Raw RNA-seq data files have been deposited in the Gene Expression Omnibus (https://www.ncbi.nlm.nih.gov/geo/) under accession GSE116752.

#### Statistical analysis

**Behavior:** SAS version 9.2 was used. Total distance traveled in the home cage on days 5 and 6 of the 6 day test during re-phenotyping was analyzed using a t-test. Distance traveled within the 2-hour period following the saline or amphetamine injection was analyzed using analysis of covariance, with the distance traveled on days 5 and 6 entered as a covariate to account for individual variation in activity levels, followed by line (High-Active or Control), and treatment (saline or amphetamine) entered as factors, and their interaction. The following criteria was used to establish normality of residual distributions: skewness between -1 and 1 and kurtosis between -2 and 2, and visual inspection of the histogram.

**Differentially expressed genes:** Differential expression analysis used the edgeR software v. 3.8.5 for R v. 3.1.2. Briefly, we filtered out any genes whose expression was not greater than 1 count per million (CPM) in at least 3 samples. The expression matrix was TMM-normalized. Using the GLM functionality of edgeR, we fitted a model corresponding to two-way ANOVA where genotype (High-Active vs. Control), treatment (saline vs. amphetamine), and their interaction were entered as factors. Dispersion was estimated using the edgeR robust methodology, which uses an admixture of the tagwise and trended dispersion estimates while calculating observational weights to be used in downstream modeling. False discovery rates (FDRs) were calculated using Benjamini-Hochberg method [22], and genes at FDR <0.10 were considered differentially expressed.

#### DAVID enrichment analysis

To test for overrepresentation of biological systems in our DEGs we used the NIAID bioinformatics annotation tool DAVID v. 6.8. The lists of upregulated and downregulated DEGs for each of the main effects and the interaction were queried. DAVID functional annotation clustering was performed using default annotation sources and a medium stringency. We used an enrichment score cutoff of >1.3 as our criterion for significant enrichment as it approximately corresponds to a p-value of <0.05.

#### Weighted gene co-expression network analysis (WGCNA)

Modules of correlated genes were identified using the WGCNA software v. 1.34 in R v. 3.0.2 [23]. Prior to coexpression analysis, we log_2_-transformed data and filtered all genes displaying zero variance. Transformation was performed using the voom+limma function in limma v. 3.22.4. We then performed WGCNA on the transformed dataset using Pearson correlation coefficients with a soft threshold power of 8, which was determined because it had good scale-free topology (R^2^>0.8), with good median connectivity (median k = 369.6), and relatively large module sizes, as done previously [21]. We ran WGCNA in signed mode using Pearson’s r for the correlation function with minimum module size set to 30, the deepSplit parameter for the cutreeDynamic function set to 2 and the mergeCutHeight parameter for the mergeCloseModules function set to 0.15. Module eigengene values were calculated for each of these modules and a two-way ANOVA parallel to the one used for the DEG analysis was used to analyze eigengene relationships genotype, amphetamine treatment, and their interaction.

### Experiment 2: Immunohistochemical detection of synaptophysin

Stereological analysis of tissue stained for synaptophysin has been used a reliable measure of synaptic numbers within the central nervous system [24–26]. When mice were approximately 3 months old, they were euthanized by transcardial perfusion with ice-cold saline, followed by chilled 4% paraformaldehyde. Brains were removed, postfixed overnight in 4% paraformaldehyde at 4°C, and then transferred to 30% sucrose solution in phosphate buffered solution (PBS) until sectioning. Brains were sectioned using a cryostat into 40 micron sections which were stored in cryoprotectant in 24-well plates and stored at −20°C.

#### Immunohistochemistry

A 1-in-6 series of sections throughout the entire rostro-caudal extent of the striatum was stained for synaptophysin using diaminobenzidine (DAB) as the chromogen to estimate relative number of synapses. All sections from all animals were treated at the same time in the same reagents using custom made immunohistochemistry trays where one tray is filled with reagents and all wells are placed in the tray together for consistent staining. Free floating sections were washed in phosphate-buffering solution (PBS) and then treated with 0.6% hydrogen peroxide in PBS for 20 min. Sections were then blocked with a solution of 0.02% Triton-X and 6% normal goat serum (NGS) in PBS (PBS-X) for 1 h, followed by incubation in primary antibody, a rabbit anti-synaptophysin (sc-17750; Santa Cruz Biotechnology, Santa Cruz, CA) at a dilution of 1:5000 in PBS-X mixed with 3% (NGS) for 24 h at 4 °C. Sections were then washed in a mixture of PBS-X and 3% NGS, then incubated in secondary antibody against rabbit made in goat (sc-2030; Santa Cruz Biotechnology, Santa Cruz, CA) at 1:200 dilution in PBS-X for 90 min at room temperature. After a wash in PBS-X without NGS sections were then treated with the ABC system (Vector Laboratories, Burlingame, CA) for 1 h, followed by a wash in a PBS-X without NGS wash buffer. Sections were then stained using a DAB kit (Sigma, St. Louis, MO). To confirm the specificity of the primary antibody, a small number of sections were processed the same way as above except omitting the primary antibody step. No immuno-labeling was detected in these negative controls.

#### Stereology

**Volume estimation:** The two dimensional area of the striatum was outlined in each of the stained sections using StereoInvestigator software. The thickness of the mounted sections was determined by measuring the depth of the focal length from the top to the bottom of the tissue using a motorized stage. Total volume of the striatum was estimated as the product of the total area of the sections by the thickness of the sections by the distances between sections.

**Density of synaptophysin particle estimation:** Density of labeled synaptophysin particles within the striatum was determined using the optical disector [27] in Stereoinvestigator. Two sections per animal corresponding to 21 and 38 from the mouse brain atlas [28] were analyzed the following way. A grid (125 x 125 μm) was placed across the sections to orient a counting frame (4 x 4 μm) in the corner of each grid square. The counting frame perimeter is made up of two “acceptance” edges and two “exclusion” edges. If a synaptophysin bouton falls entirely within the counting frame or it touches the “acceptance” edge, it is counted. All particles that contact the “exclusion” edge were omitted. The number of counted particles was divided by the total volume of the counting sites to obtain the estimate of synaptic density. Density was then multiplied by the previously calculated volume of the striatum to determine the total number of synaptophysin immunoreactive particles.

#### Statistical analysis

SAS version 9.2 was used. Total volume of the striatum, density of synaptophysin particles, and total number of particles (density multiplied by volume) were analyzed using un-paired t-test between the two groups, High-Active versus Control. The same criteria as described above was used to establish normality of residual distributions.

## Results

### Mouse behavior

As predicted, mice from the High-Active line traveled approximately 4-fold greater distances than Controls during re-phenotyping when they were 6.5 months of age, at the time when the striatal transcriptome was analyzed (t_18_ = 3.8, p = 0.001). High-Active mice traveled an average of 1.2 km/day ± 0.24 SEM, whereas Control mice traveled an average of 0.3 ± 0.03 in their cages. Analysis of distances traveled within the 2 hours following injections of either saline or 0.25 mg/kg amphetamines, indicated a significant effect of the baseline activity covariate (F_1,12_ = 139.6, p<0.0001) and significant effect of line after correcting for baseline activity (F_1,12_ = 11.8, p = 0.0049). Consistent with our previous reports [13,14], amphetamines tended to reduce activity in High-Active mice by 40%, while increasing activity in Control mice by approximately 2-fold, though the interaction was not statistically significant (F_1,12_ = 2.5, p = 0.14) given the small sample size of n = 5 per group intended for RNA-seq comparisons.

### Differentially expressed genes

A total of 14,472 genes passed the threshold of 1 CPM in at least 3 samples for sufficient expression for statistical comparisons between groups. S1 Table shows ANOVA statistics and WGCNA module membership for these genes. S2 Table shows ANOVA statistics for the WGCNA modules.

#### Genotype effect

**Fig 1:**
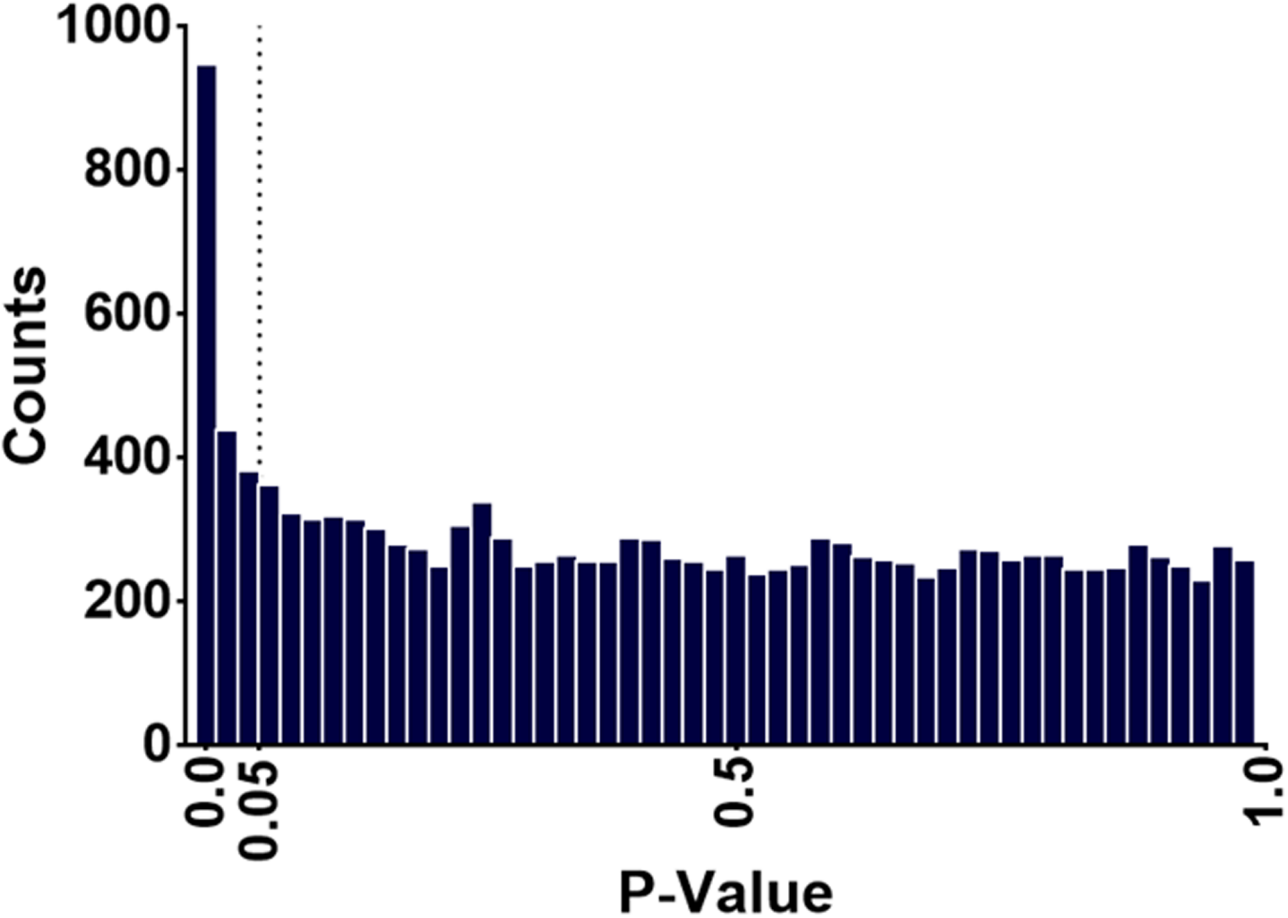
P-value distribution for differentially expressed genes in the striatum between High-Active and Control lines. Histogram of p-values for High-Active versus Control comparison of 14,473 genes expressed in the striatum.

**Fig 2:**
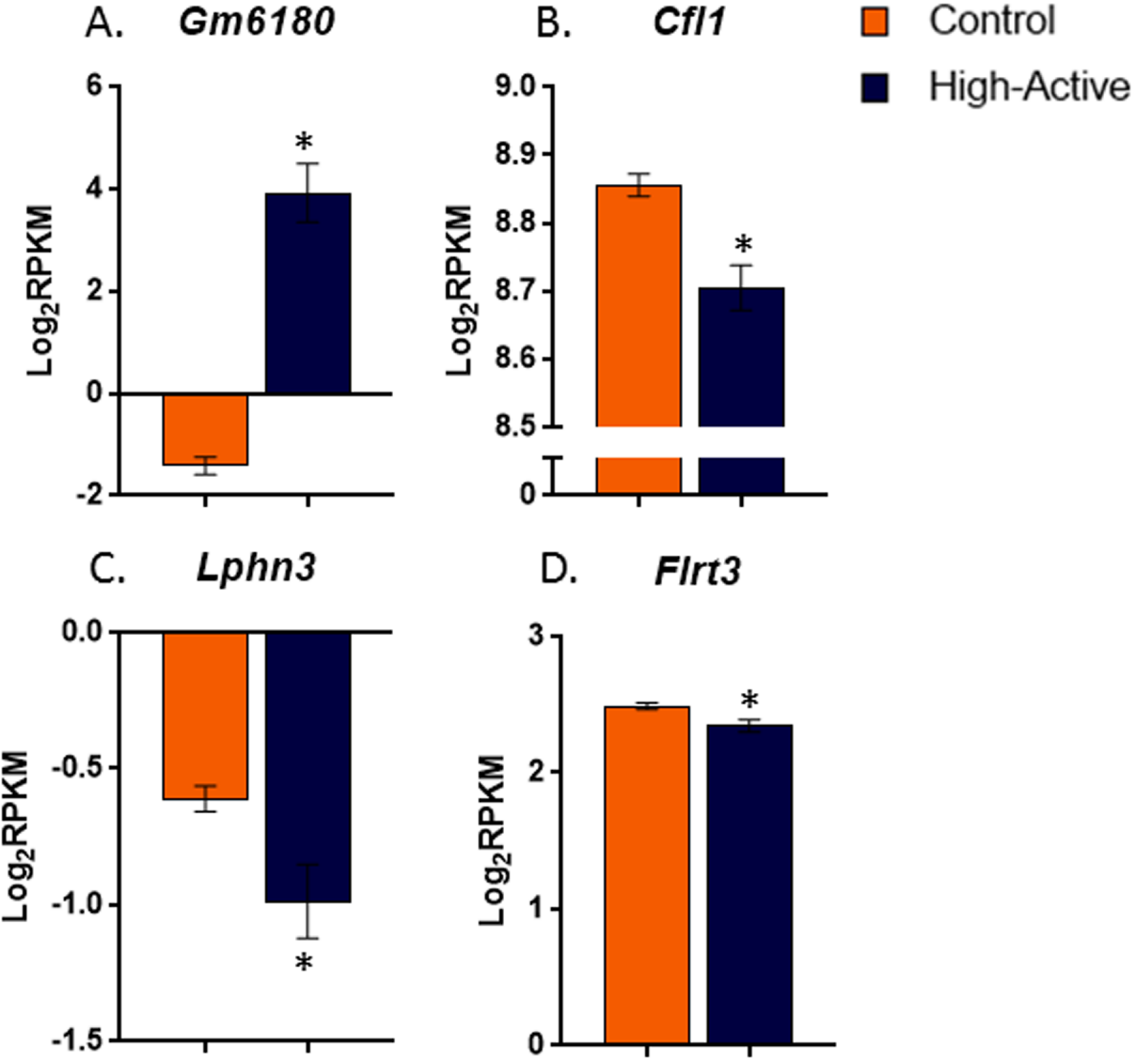
Differential expression of actin regulators and *Lphn3* related genes in the striatum of High-Active and Control mice. Average log (base 2) RPKM values (±SE) are shown separately for Control (open bars) and High-Active (shaded bars) lines (n = 10 per bar). **A**. *Gm6180*, a pseudogene for *Cfl1*, was the most significantly upregulated gene in the High-Active line. **B**. The down regulation of *Cfl1* in High-Active relative to Control may be associated with the up regulation of its pseudo gene *Gm6180*. **C**. *Lphn3* implicated in human ADHD GWAS studies [5,6] displayed decreased expression in High-Active relative to Control mice. **D**. *Flrt3*, the gene for the ligand which specifically interacts with Lphn3 to produce transynaptic scaffolding involved in synapse structure was reduced in High-Active relative to Control. *indicates significant difference using standard t test at p<0.05 level.

A histogram of the p-values comparing High-Active versus Controls shows a concentration of p-values below 0.05, suggesting that the striatum went through large scale molecular and/or physiological changes as a result of selection and/or genetic drift (Fig 1). A total of 262 genes were differentially expressed between Control and High-Active lines at FDR < 0.10 (p< 7.6 x 10^−4^), of which 141 genes were upregulated and 121 were downregulated in the High-Active relative to Control line. The top differentially expressed upregulated gene (p<3.1 x 10^−33^) was *Gm6180*, which is a non-muscle pseudogene for n-cofilin (https://rgd.mcw.edu/rgdweb/report/gene/main.html?id=735417) (Fig 2A). Expression of the *Gm6180* transcript was low in Control mice and highly expressed in High-Active mice, with an approximately 20-fold difference. These results suggest that pseudogene *GM6180* may participate in the downregulation of its parent gene *Cfl1*. Although, *Cfl1* was not significantly differentially expressed by the FDR <0.10 cut off, it was significantly reduced in High-Active relative to Controls by t-test at p<0.001 level (Fig 2B).

Latrophilin 3 (*Lphn3*) was significantly downregulated (p< 0.0004) in High-Active relative to Control (Fig 2C). The ligand of Lphn3, fibronectin leucine rich transmembrane protein 3 (*Flrt3*) was not significantly differentially expressed at FDR<0.10, but was significantly reduced in High Active relative to Control by t-test at p<0.04 level (Fig 2D). The trans-synaptic interaction between Lphn3 and Flrt3 is critical in modulating synaptic strength and density [18]; decreased levels of *Lphn3* and *Flrt3* suggest that synaptic strength and density is decreased in High-Active striatum.

**Fig 3:**
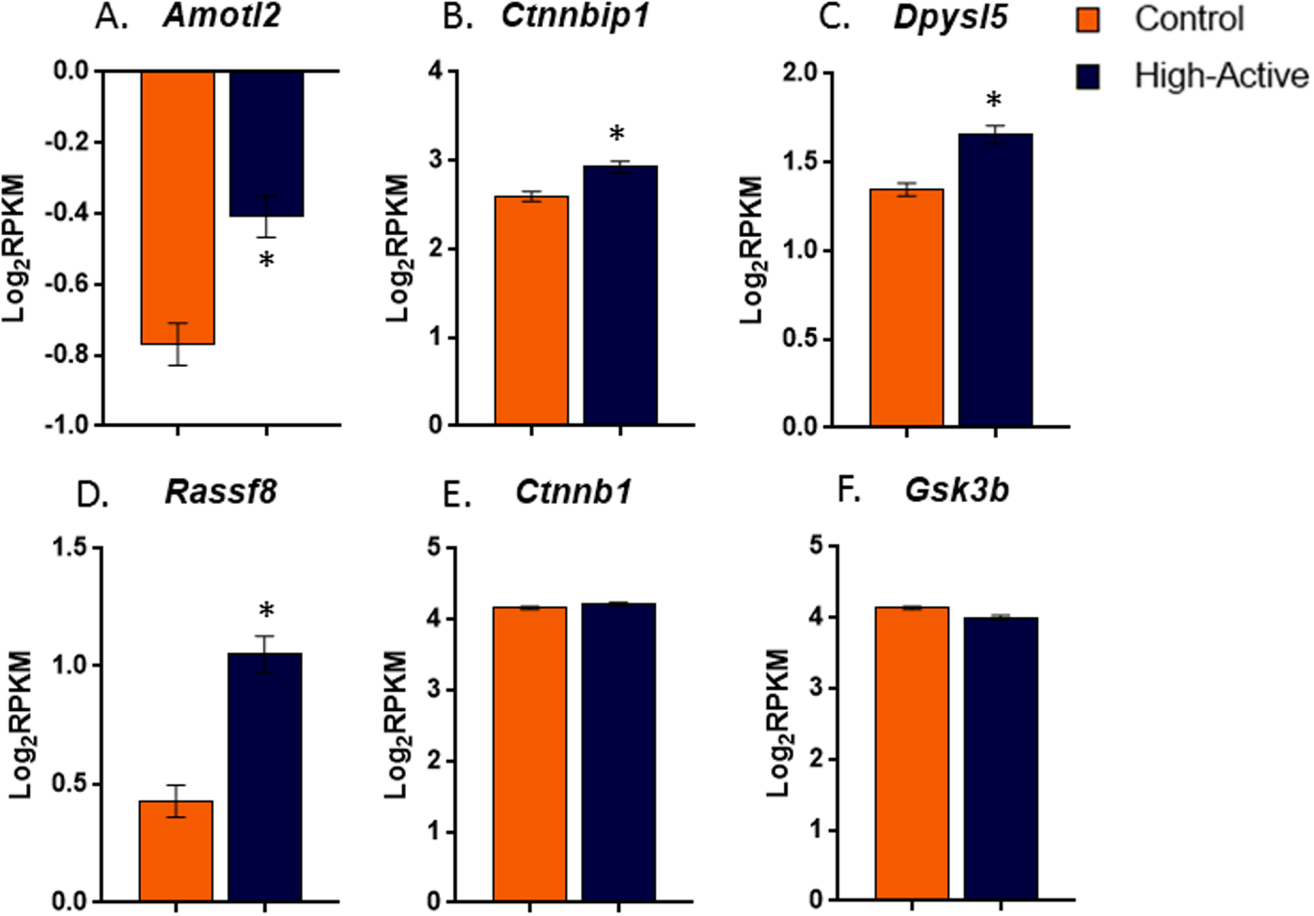
Differential expression of canonical *Wnt* pathway related genes in the striatum of High-Active versus Control mice. Average log (base 2) RPKM values (±SE) are shown separately for Control (open bars) and High-Active (shaded bars) lines (n = 10 per bar). **A-D.** *Amotl2*, *Dpysl5*, *Ctnnbip1*, and *Rassf8* were all significantly upregulated in High-Active relative to Control mice. **E-F.** *Ctnnb1* and *Gsk3β*, other genes in the canonical Wnt pathway, were not significantly differentially expressed. *indicates significant difference using standard t test at p<0.05 level.

Four canonical Wnt signaling-associated genes—dihydropyrimidinase-like 5 (*Dpysl5*), Ras association domain family member 8 (*Rassf8*), β-catenin interacting protein 1 (*Ctnnbip1*), and angiomotin-like 2 (*Amotl2*) were upregulated (all p<0.0002) in High-Active striatum relative to Control. The protein products of all four genes are known to interact with glycogen synthase kinase 3β (*Gsk-3β)* and β-catenin (*Ctnnb1)* thus modulating the canonical Wnt signaling pathway (Fig 3A–3D). Another Wnt signaling gene, transmembrane protein 44 (*Tmem44*) was also significantly upregulated in High-Active mice (p<1.4 x 10^−12^) (data not shown). Expression of canonical Wnt interacting partners, *Ctnnb1* and *Gsk-3β* were similar between the lines (Fig 3E and 3F).

#### Amphetamine and genotype-by-amphetamine interactions

**Fig 4:**
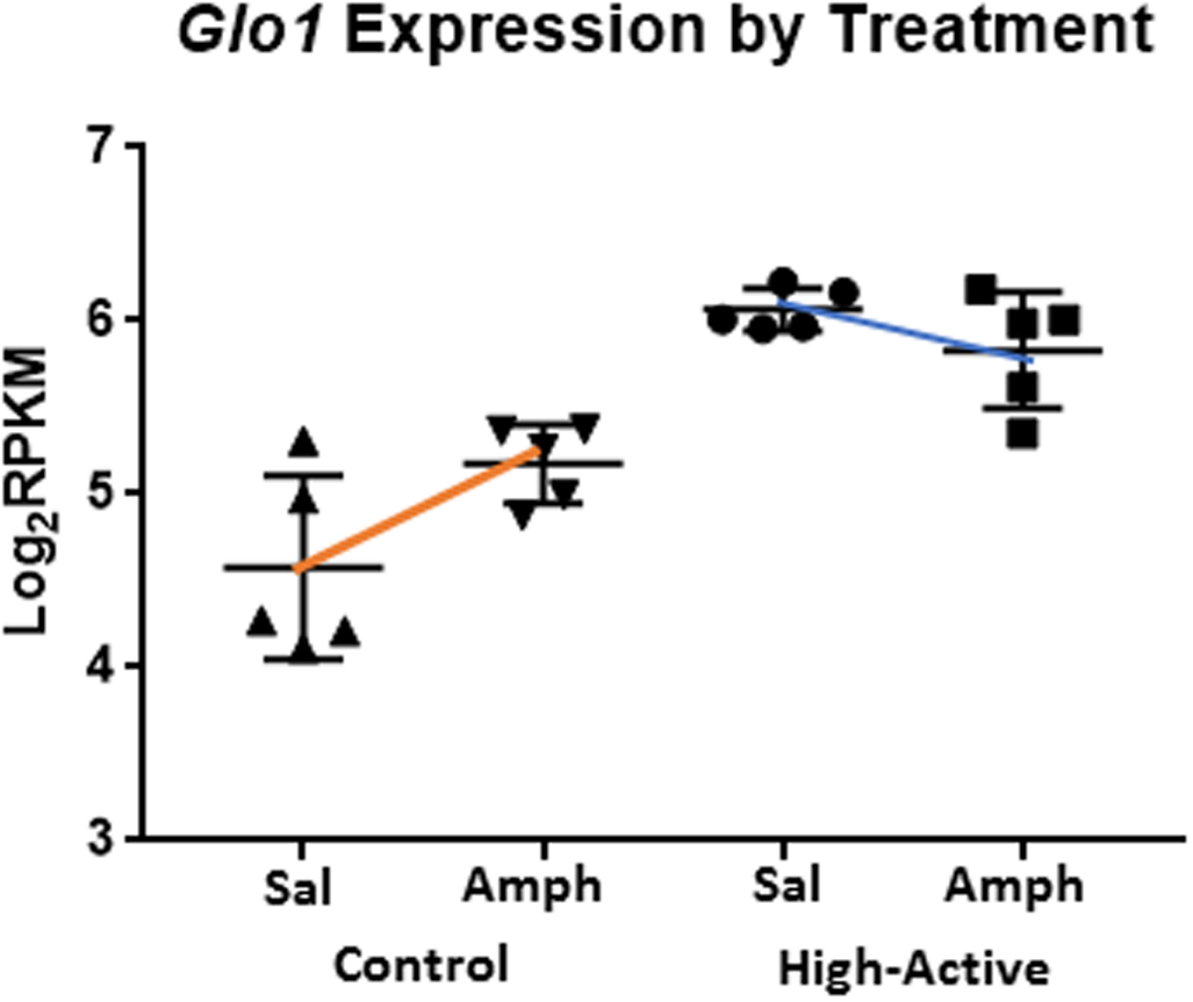
Effect of amphetamine administration on differential expression. Log (base 2) RPKM values are shown for Control-saline, Control-amphetamine, High-Active-saline, High-Active-amphetamine groups. In response to amphetamines, *Glo1* expression increased in Control mice and tended to decrease in High-Active mice.

A histogram of the p-values for the main effect of amphetamines and interaction of genotype-by-amphetamines were uniform, suggesting subtle, if any variation in gene expression was attributed to these factors. For the main effect of amphetamine, 53 genes passed the FDR < 0.10 threshold; 22 were upregulated, and 31 were downregulated. 17 genes showed a differential response to amphetamine depending on the genotype (*i*.*e*., genotype-by amphetamine interaction) at FDR < 0.10. Of the 17 interacting genes, glyoxalase 1 (*Glo1; p<1*.*6 x 10*^*−5*^) is of particular interest due to recent association with anxiety-like behaviors in mice [29]. Expression of *Glo1* was significantly higher in High-Active than Controls under both conditions, but amphetamines tended to reduce *Glo1* expression in High-Active whereas it increased expression in Controls (Fig 4).

### DAVID enrichment analysis

Among the 121 genes significantly downregulated in the High-Active line, functional categories related to lipid biosynthesis, oxidoreductase activity, and PPAR signaling were significantly enriched with scores of 1.67, 1.59, and 1.59 respectively. Among the 141 genes significantly upregulated in the High-Active line, categories associated with ATP-grasp fold and prenylation were significantly enriched with scores of 1.94 and 1.43, respectively.

### WGCNA

Following Saul et al. [21] the analysis of all genomes in the genome annotation which had non-zero variance (26,114) in WGCNA yielded 49 modules with a maximum module size of 2536 genes and a minimum module size of 84 genes. WGCNA was unable to cluster 2463 genes, which were clustered into module 0. Three modules (numbered 8, 14, and 44) showed significant differences between the genotypes at FDR<0.10 (p<0.002) (S2 Table). Module 8 included *Flrt3* and other genes overrepresented for cytoskeletal proteins (DAVID enrichment score: 2.42), mitochondrial activity (DAVID enrichment score: 4.05), DNA damage repair (DAVID enrichment score: 3.13). Module 14 included genes for overrepresented ontologies such as pyridoxal phosphate activity (DAVID enrichment score: 1.80) and proteolysis (DAVID enrichment score: 1.65). Module 44 included genes overrepresented for actin-myosin complex formation (DAVID enrichment score: 1.95), Golgi apparatus (DAVID enrichment score: 1.76), and ATP binding (DAVID enrichment score: 1.40). None of the modules displayed significant main effect or interaction with amphetamines. Results of the DAVID analysis on the three significant modules are shown in S3 Table.

### Immunohistochemical detection of synaptophysin

**Fig 5:**
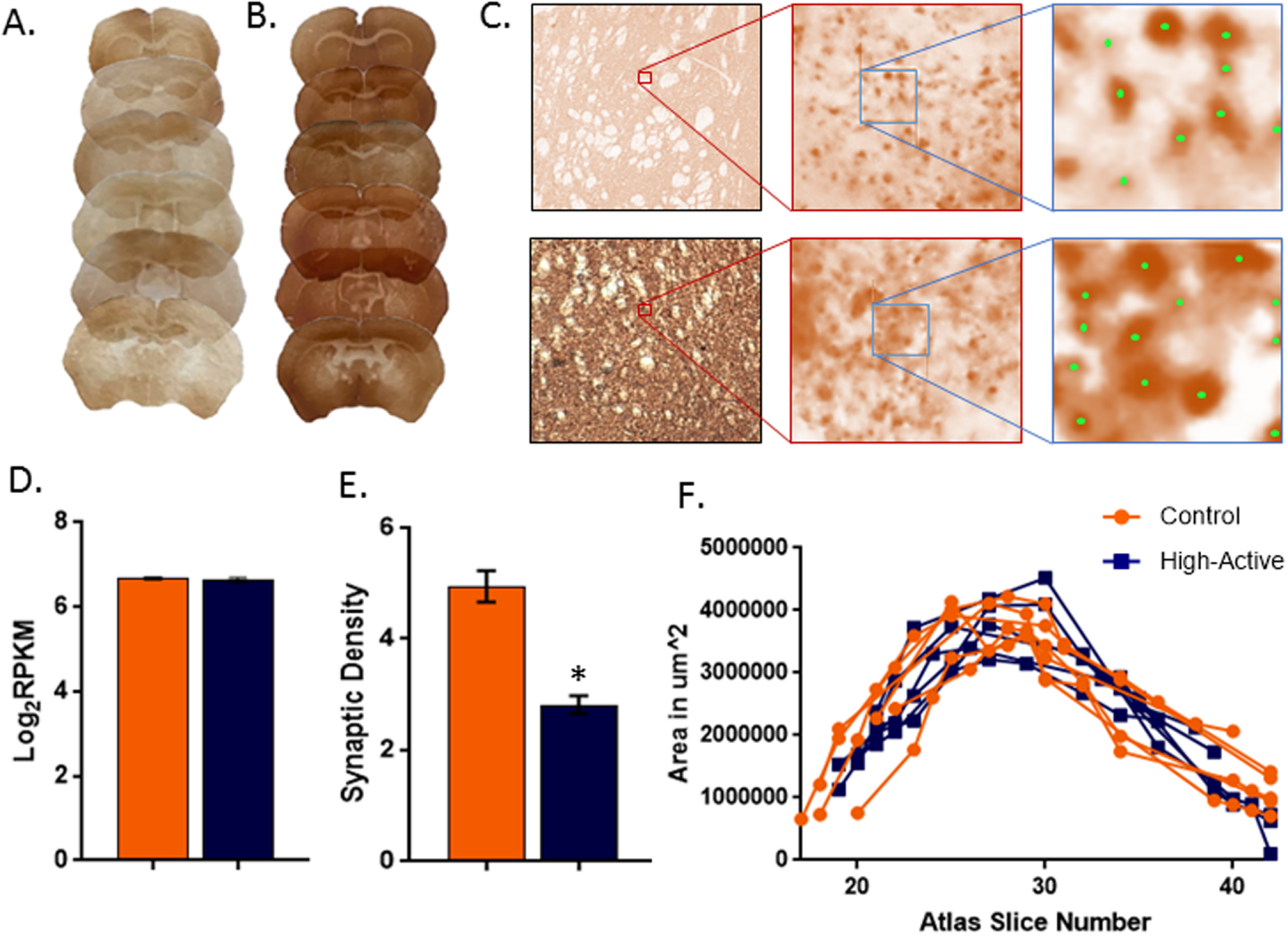
Reduced synaptophysin labeling in High-Active relative to Control mice. **A.** Representative sections from High-Active (left column) and Control (right column) mice. A section from each mouse in the study is shown at different positions along the rostro-caudal axis. Zoomed in images of **B.** High-Active and **C.** Control striatum at 2.5x magnification and 100x magnification, with a further zoomed in image of the counting frame with sample counts (green). Left and bottom boundaries of the counting frame are exclusion boundaries, and top and right are inclusion boundaries. **D.** Average synaptophysin gene expression in the striatum from the RNA-seq dataset showing no difference between genotypes. **E.** Mean (± SE) density of synaptophysin positive particles (number of particles x10^7^ per mm^3^) in High-Active and Control mice showing significant reduction in High-Active. **F.** Unilateral, two-dimensional areas of the striatum plotted against section number (based on the mouse brain atlas [28]) for each animal separately showing no difference between genotypes.

The density of synaptophysin positive particles was significantly reduced in High-Active relative to Control mice, by approximately 44% (t_10_ = 8.8, p<0.0001; Fig 5A–5C and 5E). The volume of the striatum did not differ and was estimated to be 15.1 cubic mm (± 1.11 SE) and 14.5 cubic mm (± 0.97 SE), in Control and High-Active mice, respectively (Fig 5F). The total number of synaptophysin particles (the product of density and volume) was also significantly reduced in High-Active relative to Control by approximately 46% (t_10_ = 9.8, p<0.0001). In High-Active mice the total number of synaptophysin particles was estimated to be 4.0 x 10^8^ (± 0.11 SE) particles per cubic mm, whereas in Control mice the estimate was 7.4 x 10^8^ (± 0.18 SE).

## Discussion

The main finding of the study is the robust molecular signature of ADHD-like behavior in the striatum. Rather than the usual suspects for the catecholamine hypothesis of ADHD, such as monoamine transporters and receptors, results identify new candidate genes and confirm others. Three converging pathways, synaptic actin regulation, Latrophilin 3, and canonical Wnt all suggested a downregulation in maintenance and regulation of excitatory synapses. This molecular prediction was confirmed by immunohistochemical detection of synaptophysin, a synaptic vesicle protein marker widely used as a marker of synapses [24]. High active mice displayed approximately 46% reduction in number of synapses compared to Control. Taken together with recent findings from animal models and human GWAS studies, results support the hypothesis that ADHD-like behavior is associated with changes in multiple genes and pathways that may ultimately reduce or impair excitatory synapses in the striatum.

### Synaptic actin regulation

Pseudogenes often negatively regulate their parent genes [30]. Therefore, it is possible that the upregulation of *Gm6180* exerted a negative influence on *Cfl1* that resulted in *Cfl1* being downregulated in High-Active mice (Fig 2A and 2B). However, this hypothesis requires additional molecular work before it can be confirmed. A role for *Cfl1* in synaptic remodeling of the striatum and predisposition for ADHD-like behavior was recently demonstrated in mutant mice with *Cfl1* and actin-depolymerizing factor (*ADF)* knocked out specifically in the striatum [31]. *Cfl1* was deleted in the striatum by controlling expression through a CaMKII-Cre transgene (*ADF*-/*Cfl1*- mice). These mice displayed abnormal synaptic structure and decreased excitatory synaptic density. In parallel with the synaptic alterations, the mice displayed ADHD-like symptoms of hyperactivity and impulsivity which were ameliorated with methylphenidate [31]. The *ADF*-/*Cfl1*- mice and our High-Active mice share remarkable convergence in striatal *Cfl1* expression and behavioral phenotypes related to ADHD. If the *Gm6180* interaction with *Cfl1* is indeed true, then *Gm6180*-like interactions may represent novel targets for modulating synapse strength in the striatum with specificity for ameliorating ADHD-like symptoms.

### Latrophilin 3

Decreased *Lphn3* and *Flrt3* expression in High-Active versus Control striatum (Fig 2C and 2D) provides yet further evidence in support of the hypothesis that reduced excitatory synapses in the striatum predispose ADHD-like behavior in the model. The trans-synaptic interaction between the protein products of *Lphn3* and *Flrt3* is of particular relevance due to the fact that genetic variants in *Lphn3* have been associated with ADHD in GWAS studies of large human cohorts [6]. In particular, SNP marker rs6551665 is associated both with susceptibility to ADHD and with response to stimulant medication such as amphetamine and methylphenidate [15]. Further, *Lphn3* null mutant mice display ADHD-like phenotypes such as hyperactivity, differential sensitivity to cocaine, and alterations in whole-brain levels of dopamine and serotonin-related genes and amino acid levels [17]. Our results support our hypothesis that downregulation of *Lphn3* along with the endogenous ligand, *Flrt3*, contribute to fewer or abnormal excitatory synapses in the striatum, which contributes to the ADHD-like symptomology.

### Canonical Wnt pathway

**Fig 6:**
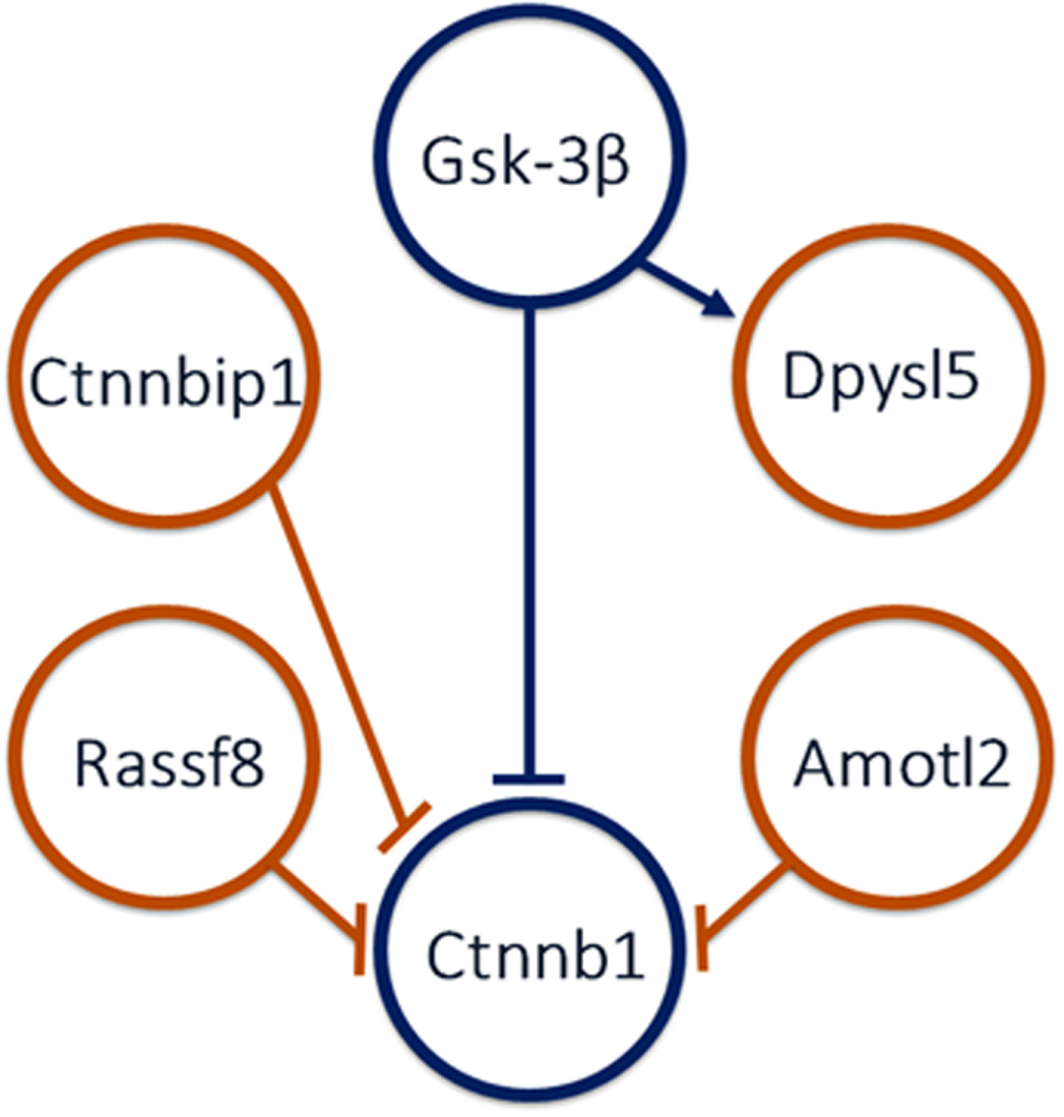
Hypothesized interactions between canonical Wnt-associated proteins. A synthesis from the literature of known protein interactions in the Wnt pathway for significantly differentially expressed genes between lines is shown. Orange indicates the gene for the protein was upregulated in the High-Active relative to Control mice. Blue identifies canonical Wnt proteins. Lines extending from one node toward another indicate the former regulates the latter. Arrows indicate positive regulation, whereas terminals (flat, perpendicular ends) indicate negative regulation.

Of the 262 genes differentially expressed between lines, 5 are associated with the canonical Wnt pathway. Three of these genes, *Ctnnbip1*, *Amotl2*, and *Rassf8* encode proteins that inhibit the function of the Ctnnb1 protein [16,32,33] (Fig 6), suggesting that the canonical Wnt pathway could be disrupted in the striatum of High-Active mice. *Dpysl5* (also known as *Crmp5*) and *Tmem44* were also upregulated in High-Active mice and have known interactions with the canonical Wnt pathway, though the functional directionality of the interactions is not as clear [34,35] (Fig 6).

The over-representation of canonical Wnt pathway genes that are significantly differentially expressed in a manner consistent with downregulation of the Wnt pathway suggest that modulation of Wnt signaling in the striatum is central to the High-Active phenotype. Recent evidence has shown that variation in Wnt signaling is a hallmark of other developmental psychiatric disorders, particularly schizophrenia, bipolar disorder, and autism spectrum disorder [36]. Specifically, key Wnt signaling downregulators have been associated with populations with schizophrenia and bipolar disorder [37–40]. Drugs that are used to treat both disorders are also known to raise Ctnnb1, a Wnt signaling biomarker, in the brains of adult rats. Amphetamine doses that are known to induce psychosis also decrease levels of Ctnnb1, but the effect of therapeutic doses of amphetamines on Wnt signaling in an ADHD-like brain, to the best of our knowledge has not been explored [41,42].

The canonical Wnt pathway is crucially involved in synapse formation during brain development and maintenance through cell-to-cell adhesion processes, neuronal formation, and proliferation/migration [43,44]. In the adult brain, Wnt signaling functions in synaptic cytoskeleton stabilization [45]. Downregulation of Wnt signaling could have many implications, ranging from potential decreases in synaptic density, abnormal synapse formation, and/or impaired cell-to-cell adhesion. *Dpysl5*, a member of the Wnt pathway upregulated in our results, is specifically implicated in controlling neurite outgrowth [34]. Coincidentally, altered actin dynamics due to decreased levels of Cfl1 and trans-synaptic binding molecules described above were predicted to cause similar effects on the synapse [31]. Taken together, these results suggest multiple molecular pathways are at play in the High-Active mouse striatum which converge on synapse formation and maintenance.

### Glyoxalase 1

The vehicle-treated High-Active mice displayed approximately three-fold higher expression of glyoxylase1 (*Glo1)* relative to Control, while amphetamine reduced expression. This response was opposite to Control mice, where amphetamine increased expression (Fig 4). As reviewed by Distler & Palmer (2012), *Glo1* is implicated in anxiety-related behavior in mice [29]. GLO1 is an enzyme involved in cellular digestion of glucose, metabolizing and reducing cellular levels of the byproduct methylglyoxal (MG), primarily in astrocytes as opposed to neurons [46]. MG is a GABA-A receptor agonist and infusion into mice reduces anxiety-related behaviors. This is interesting because the majority of striatal neurons are GABAergic projecting medium spiny neurons. It is possible that the high baseline levels of *Glo1* in the High Active line cause low MG levels which reduce GABAergic signaling in the striatum, but how this causes anxiety, and how an anxiety phenotype might be related to increased physical activity and impulsivity in our lines remains to be determined.

### DAVID

The most significantly enriched category among genes downregulated in High-Active mice involves lipid biosynthesis. Most of the brain is composed of lipids, and lipids serve many different functions in the brain, and so it is difficult to interpret this generic result. Low serum levels of fatty acids have been observed in populations with ADHD regardless of diet [47–49]. It is possible that lipid synthesis dysregulation occurs in the brain as well as other organs but connections to ADHD symptomology remains unclear.

The peroxisome proliferator-activated receptor (PPAR) pathway is associated with lipid metabolism [50], and is also significantly enriched in genes downregulated in High-Active mice. PPARγ inhibits Gsk3β [51], and Gsk3β inhibits Ctnnb1 (Fig 6). Hence, decreased PPARγ inhibition of Gsk3β in High-Active striatum would result in greater inhibition of Ctnnb1, consistent with the hypothesized decrease in Wnt signaling in High-Active striatum.

ATP-grasp fold enzymes and prenylation associated genes are enriched in the upregulated genes in High-Active mice. ATP-grasp fold enzymes are involved in fatty acid synthesis [52]. We speculate that ATP-grasp fold enzymes and related pathways may be upregulated in High-Active mice as a compensatory mechanism to counterbalance decreased lipid metabolism and biosynthesis. Post-translational prenylation plays a role in protein localization and interactions [53], and thus could be involved in a variety of processes that have not been elucidated in this model yet.

### WGCNA

Module 8 was most significantly different between the lines, and over-represents genes associated with cytoskeleton proteins, including *Flrt3*. The implication is that in addition to *Gm6180-Cfl1* and *Lphn3*-*Flrt3*, there are many other process that likely contribute to altered cellular and synaptic structure in the High-Active striatum. Module 14 contains *Tmem44 and Glo1* as well as other genes associated with pyridoxal phosphate activity and proteolysis. We know of no current consensus about the involvement of pyridoxal phosphate and proteolysis in ADHD or other developmental disorders.

Module 44 over-represents genes associated with the actin-myosin complex and ATP binding. We would expect genes associated with other processes involving actin, such as the actin-myosin complex, to be dysregulated, in line with our synaptic actin hypothesis. The overrepresentation of genes associated with ATP binding also relates to the DAVID result involving ATP-grasp fold genes being upregulated in High-Active mice.

### Reduced synaptophysin labeling in High-Active mice

The molecular prediction that synapse number would be reduced in the striatum of High-Active mice was confirmed in a separate set of animals processed for immunohistochemical detection of synaptophysin. The difference was striking, strongly supporting the hypothesis that the molecular changes which promote hyperactivity and impulsivity in the High-Active line do so, in part, by decreasing the number of synapses in the striatum (Fig 5A–5C). All the sections from all the animals were treated using the same reagents at the same time (see Methods), thus the large difference in staining is a real biological difference rather than a batch effect from the immunohistochemistry. It is important to point out that synaptophysin gene expression was similar between High-Active versus Control in the striatum (Fig 5D), suggesting that synapses were reduced not by decreasing their rate of formation but rather by altering their regulation, maintenance and stability. However, as synaptophysin does not distinguish between excitatory or inhibitory synapses, further work is needed to identify the specificity in the types of synapses that are reduced in High-Active mice. Previous work done by Zimmerman et al. [31] showed a 16% decrease in excitatory synapses in mice engineered to display reduced Cfl1 in the striatum, and the High-Active mice displayed reduced Cfl1 (Fig 2B). Human ADHD has been associated with genetic variants in synapse-related genes [54] including synaptophysin [55]. The genetic association between *Lphn3* and ADHD in human populations has been suggested to result from the allele’s effects on synapse stabilization. Lphn3 is thought to play a role cell adhesion and trans-synaptic scaffolding [18] and *Lphn3* was reduced in the High-Active mice (Fig 2C). Reduced excitatory synapses is consistent with reduced gray matter [56] and hypo-activation of the striatum observed in ADHD [57]. Future work with the High-Active mice could reveal molecular pathways connecting genetic association between synapse development-related genes such as *Lphn3*, synapse maintenance and ADHD-like behavior.

### Limitations

RNA was extracted from a large portion of the dorsal and ventral striatum which includes a mixture of multiple different cell types (e.g., neurons, glial cells and endothelial cells). Hence, differences in gene expression between groups in our study could be driven by changes occurring in one or multiple of these cell types, as we did not differentiate the pathways at the cellular level. The converging evidence points to synapse maintenance and regulation, so it is likely that neurons and astrocytes were the main cell types influencing the gene expression results emphasized. In addition, because we used both dorsal and ventral striatum, we could have missed genes significantly expressed in the dorsal striatum but not the ventral and vice versa, by homogenizing these two distinct regions. Future analyses of samples containing isolated cell types is required for confirmation.

The gene expression differences between the High-Active and Control lines in our study could be due to selection for the ADHD phenotype, but could also be due to random genetic drift. The two lines were reproductively isolated for 17 generations, with a relatively small effective population size (approximately 10 families per line). One way to remove this confound in future studies is to develop and analyze additional replicate lines of mice [21]. It is alternatively possible to estimate rates of genetic drift without replicate lines if certain assumptions are made about heritability, effective population size, and inbreeding coefficients [13,14].

Because the High-Active line was generated through selective breeding, it can safely be assumed that the observed differences in behavior and gene expression relative to the Control line can ultimately be attributed to specific allelic differences that occur at multiple places in the genome between the lines. However, no QTL or gene mapping data are available for these lines, and the RNA sequence data obtained from this dataset is not adequate for statistically establishing genetic associations. In the present study, some of the DEGs between the lines were transcription factors (*e*.*g*., *Preb*, *Nkx6-2*, *Hopx*, S1 Table), which presumably contributed to orchestrating the gene expression signatures of the lines. Future work employing such techniques as chromosome immuno-precipitation sequencing (ChIP-seq) could identify DNA sequence variation at the site of interaction between the DNA binding molecules and the DNA, that ultimately could explain the gene expression regulation.

### Conclusions

Results establish substantial molecular reorganization of the striatum in response to 17 generations of selection for hyperactivity. Three pathways were identified that together converge on a story of abnormal synaptic structure and maintenance. As of yet, no known interactions between the three main identified pathways, canonical Wnt, actin depolymerization, and *Lphn3* have been established. These may represent independent mechanisms or complement and support each other. The mechanism underlying the negative association between *Gm6180* and *Cfl1* expression could provide a useful molecular target if it proves capable of altering synapses in a subtle and specific way in the striatum to predispose ADHD. In addition, DAVID analysis suggests a broader dysregulation of lipid biosynthesis in High-Active mice, in line with current human ADHD literature. Future plans include quantifying excitatory synaptic density, dendritic spines, and arborization in High-Active vs. Control striatum to directly test and refine the reduced excitatory synapse hypothesis motivated by the gene expression and synaptophysin results highlighted herein. We hope by unraveling the biology, from the molecular regulators to the brain-circuit level physiological outcomes, it may be possible to one day identify better targets at the root of the pathophysiology of ADHD.

## Supporting information

S1 Table Differentially expressed genes. “Selection” refers to effect of selection, “drug” refers to effect of amphetamine, and “interaction” refers to the interaction between “selection” and “drug” in a 2-way ANOVA of log RPKM. “repheno” refers to the correlation between gene expression and distance traveled on days 5 and 6 at the 6.5 month time-point. “drug.response” refers to correlations between gene expression and distance traveled on day 7 within the 2 hour period after the mice received the saline injection or the amphetamine injection up until the point when they were euthanized. “pnd60.activity” refers to correlation between gene expression and distance traveled at the 3 month time-point during original behavioral phenotyping. “logFC” refers to log fold change for categorical variables; “cor” refers to correlations for continuous variables. “LR” refers to likelihood ratio, “logCPM” gives log of counts per million. All other column labels use standard acronyms.
(XLSX)

S2 Table WGCNA modules. See S1 Table.
(XLSX)

S3 Table WGCNA DAVID results. DAVID enrichment analysis results for WGCNA modules 8, 14, and 44. These modules’ eigengenes showed significant effects of selection.
(XLSX)
